# Supplementary material for: pdb-tools: a swiss army knife for molecular structures
Source: F1000Res. 2018 Dec 20;7:1961. [Version 1] doi: 10.12688/f1000research.17456.1 (PMC6343223; doi:10.12688/f1000research.17456.1)
Supplement: Peer review history

# Peer review: Referee response for version 1

**DOI:** 10.5256/f1000research.19090.r42177

**Published:** 2019-01-18

**Authors:** Peter W. Rose

**Affiliations:** 1 Structural Bioinformatics Laboratory, San Diego Supercomputer Center, University of California, San Diego, La Jolla, CA, USA

While there are many, often complex, tools available to manipulate pdb files, pdb-tools stands out as a light-weight set of command line tools for common use cases. The requirement for each tool to perform one task makes these tools easy to use in shell scripts and to combine into a pipeline for batch processing a set of PDB structures.

 On the software side, this is a well-designed open source tool kit available in GitHub that follows best software development practices, including documentation, checking for consistent coding style, high test coverage, using continuous integration, semantic versioning, and deployment on PyPi. A liberal open source license encourages community contributions. The modular design makes this project easy to maintain and expand compared to many of the legacy tools still in use.

 Recommendation:

 List all, not just a subset, of the available tools with an example on the documentation page. That way, a Google search will find those tools and examples are helpful for new users.

I have read this submission. I believe that I have an appropriate level of expertise to confirm that it is of an acceptable scientific standard.

# Peer review: Referee response for version 1

**DOI:** 10.5256/f1000research.19090.r42178

**Published:** 2019-01-04

**Authors:** Bruno L. Victor

**Affiliations:** 1 BioISI, Biosystems and Integrative Sciences Institute, Department of Chemistry and Biochemistry, Faculty of Sciences, University of Lisbon, Lisbon, Portugal

In this work, Rodrigues et al, present to the readers a collection of python based tools to edit, convert and validate PDB files, in a very simple and efficient way. The authors clearly describe in the methods section the procedure used to implement the pdb-tools toolkit, and they even provide some simple use cases where the toolkit can be applied. I have installed the toolkit in my workstation and I did not have any kind of problem. I have tested several of the available functionalities and I have found it to be very useful and easy-to-use on day-to-day work (as the authors clearly state in the article). This type of contribution software is very useful for any scientist (with beginner or advanced skills) dealing with structure data in PDB format.

I have read this submission. I believe that I have an appropriate level of expertise to confirm that it is of an acceptable scientific standard.
